# Presidential Address

**Published:** 1882-11

**Authors:** 


Address Delivered by the President of the Homœopathic Medical Society of Pennsylvania, John C. Morgan, M. D., at the 18th Annual Session, held at Altoona, Sept., 1882.

Dr. Morgan's address is a very interesting one and far superior to the trash usually sent forth in "presidential addresses." We comment on a few points. After discussing Hahnemann's declaration that *the sole duty of the physician is to heal the sick,* Dr. Morgan adds: "The American general practitioner of to-day cannot be limited to Hahnemann's dictum. He cannot always be the physician; and as surgeon, he must primarily contemplate his duties from an entirely new point of view." That is a queer assertion; if it be not the surgeon's duty to heal the sick, what is his duty?—*to kill?*

"No one is *purely* a 'physician;' hence, no one can be absolutely hedged in, in his duties, by this initial dictum," says Dr. Morgan. We deny this, and assert that as far as the relations of physician and patient are concerned the doctor is only *employed* to heal the sick, whether he does this by means of drugs, hygiene, or the mechanical methods of obstetrics and surgery. Homœopathy is superior to the knife, as Dr. Morgan admits: "Hahnemann and his immediate disciples, however, were accustomed to rescue so many cases from surgery—cured so many ulcers, tumors and other so-called surgical cases with medicine only—that they held the surgery of their day in deserved contempt."

After reviewing Hahnemann's "theories," Dr. Morgan concludes as follows:

"We have, then, no reason to be ashamed of or to discard 'the theories of Hahnemann.' The God of Nature spoke to that venerable sage. Hahnemann bowed to the inspiration, and gave us the *Organon* of Homœopathy. We may well lay aside apologies and follow so illustrious an example. We may well be proud of him, at all points; and of one another, as the disciples of a seer who, when the discoverers of these facts were as yet in their cradles, not only gave to the world the principles of Homœopathy, but reduced all of them to successful practice. Truly, we are partners in a goodly heritage."

